# Berberine-loaded albumin nanoparticles reverse aflatoxin B1-induced liver hyperplasia

**DOI:** 10.1186/s40360-023-00683-w

**Published:** 2023-08-10

**Authors:** Sarah M. Khedr, Doaa A. Ghareeb, Shadia A. Fathy, Germine M. Hamdy

**Affiliations:** 1 Department of Biochemistry, Faculty of Science, Ain Shams University, Cairo, 11566 Egypt; 2 Bio-Screening and Preclinical Trial Lab, Department of Biochemistry, Faculty of Science, Alexandria University, Alexandria, Egypt

**Keywords:** Desolvation method, Cell death, Apoptosis, Insulin growth factor 2, Arginase-1, Cyclooxygenase-2

## Abstract

Hepatocellular carcinoma (HCC) can be produced from aflatoxin B1 (AFB1) administration. Although berberine (BER) acts as an anticancer agent and can counteract the AFB1 effect, it has low bioavailability. Nanotechnology can overcome this problem. This research aimed to synthesize berberine nanoparticles (NPs) and then estimate their therapeutic effect compared to that of berberine against aflatoxin-induced hepatotoxicity. The desolvation method was used to prepare BER–NPs. Aflatoxicosis was induced by 5 consecutive intraperitoneal injections (IP) of 200 µg/kg/day AFB dissolved in dimethylsulfoxide (DMSO). After the induction period, two treatments were performed: the first with 100 mg/kg BER and the second with 10 mg/kg BER-NPs. Liver, kidney, and diabetic profiles were estimated by using standardized methods. Hepatic oxidative stress, inflammatory, cancer cell proliferation, and invasion markers were used by ELISA and qPCR techniques. The TEM image shows that both BSA NPs and BER-BSA NPs had spherical, regular, and uniform shapes. The BER encapsulation efficiency % was 78.5. The formed-BER-BSA NPs showed a loading capacity % of 7.71 and the synthesis yield % of 92.6. AFB1 increases pro-oxidant markers, decreases antioxidant systems, stimulates inflammatory enzymes, inhibits anti-inflammatory markers, decreases tumor suppressor enzymes, increases oncogenes, increases glycolytic activity, prevents cell death, and promotes cell growth. Most of the biochemical markers and hepatic architecture were normalized in the BER-BSA NP-treated group but not in the BER-treated group. Altogether, the obtained data proved that treatment with BER-NPs was more efficient than treatment with berberine against aflatoxicoses induced in rats.

## Introduction

Hepatocellular carcinoma (HCC), which is one of the major types of primary liver cancer, is one of the ten most common cancers worldwide [1]. There are several factors that lead to the HCC incident, one of which is the consumption of food contaminated with aflatoxin B1 (AFB1), which is a poisonous by-product of the soilborne fungus *Aspergillus*, which is responsible for the decomposition of plant materials [2]. In hepatocytes, AFB1 is converted to reactive 8,9-epoxide by the action of the CYP450 enzyme isoforms CYP3A4 and CYP1A2, which are responsible for the formation of DNA adducts and consequently lead to the development of HCC. AFB-BNA adduct formation is positively correlated with vascular endothelial growth factor (VEGF) expression, which indicates that AFB1 induces angiogenesis and apoptosis signaling through mutations in p53 [3]. Moreover, AFB1 induced oxidative stress, which inhibited the PI3K/AKT/mTOR pathway and promoted autophagy [4]. Berberine (BER; C_20_H_19_NO_5_) is a derivative of the isoquinoline alkaloid compound. It is found in the root, rhizome, stem, fruit, and bark of various species of plants [5]. It has several pharmacological actions, including antimalarial, antiarrhythmic, hypoglycemic, hypolipidemic, hepatoprotective, antioxidant and antimicrobial activities. Anti-inflammatory and anticancer effects. Therefore, BER formulations are used in the treatment of many inflammatory conditions, diarrhea, insulin resistance (IR), neurodegeneration and liver diseases [6]. Berberine acts as an anticancer agent by several mechanisms, where it suppresses c-MYC and prevents cancer cell proliferation, upregulates p53, inhibits the cell cycle and activates mitochondrial apoptosis [7].

Despite the importance of these BER, it is classified as the Biopharmaceutics Classification System 3 (BCS 3), which means that it has low permeability, as indicated by its low oral bioavailability, which is less than 1%. Thus, for beneficial therapeutic activity, a high dose of BER (100–250 mg kg^−1^ day^−1^ for animals, 900–1500 mg day^−1^ for humans) is needed [8]. Unfortunately, a high dose of BER leads to several side effects, such as anorexia, stomach upset, diarrhea or constipation. Therefore, nanobased formulations are believed to be ideal candidates to increase the percentage of absorption, as at the nanoscale level, compounds can absorb rapidly in the gut [9].

Nanotechnology is a somewhat new part of science that has found many uses ranging from energy creation to modern creation cycles to biomedical applications. One of the critical applications is in science and biomedical exploration [10]. Nanoparticles (NPs) can be designed to have extraordinary arrangements and functionalities, giving novel apparatuses and methods that have not recently existed in biomedical exploration. For example, NPs can be utilized to depict natural cycles at the cellular level. They can also be used to recognize analytes in the attomolar range. There are many kinds of NP stages with contrasting sizes, shapes, creations, and functionalities. Moreover, each type of NP might be manufactured utilizing various methods, such as nanoprecipitation and lithography, for polymeric NPs.

Therefore, the main objective of this research was to increase the bioavailability of berberine by synthesizing berberine nanoparticles and to estimate its therapeutic effect compared to that of berberine against aflatoxin-induced hepatotoxicity.

## Materials and methods

### Materials

Berberine chloride, AFB1, bovine serum albumin (BSA), thiobarbituric acid (TBA), glutathione (GSH) and glutaraldehyde were purchased from Sigma-Aldrich (USA). HPLC-grade ethanol was obtained from Fisher Chemicals (USA). Routine blood parameter kits for glucose, triglycerides (TG), cholesterol, alanine transferase (ALT), aspartate transferase (AST), alkaline phosphatase (ALP), lactate dehydrogenase (LDH), gamma-glutamyl transpeptidase (GGT), total protein (TP), albumin (ALB), total bilirubin (T. Bil), alpha-fetoprotein (AFP), and C-reactive protein (CRP) were purchased from Spectrum, Egypt. Inducible nitric oxide synthase (iNOS), cyclooxygenase-2 (COX-2) and arginase were purchased from INOVA, China. For qPCR, primers were synthesized by Thermo Fisher Scientific (USA). iNtRON Biotechnology (Korea) was used to obtain the Easy redTM total RNA extraction kit, as well as the cDNA synthesis kit and RealMOD™ Green w2 2X qPCR mix kit. Other chemicals were obtained with high grades.

### BER-BSA NP preparation and characterization

Drug-free bovine serum albumin (BSA) nanoparticles and berberine-loaded nanoparticles cross-linked with glutaraldehyde were prepared using the desolvation method [11]. The ratio of BSA to BER was 10:1, and the amount of crosslinker was 118 μL of an aqueous glutaraldehyde solution (8%). The nanoparticles were collected by centrifugation at 20,000 rpm at 4 °C for 30 min. The free BER in the supernatant was determined by using an ultraviolet–visible (UV–Vis) spectrophotometer at 344 nm, and then the BER encapsulation efficiency (EE%), loading capacity (LC%) and NP synthesis yield (SY%) were calculated using equations EE (%) = [Initial BER (mg) – Free BER in supernatant (mg)/Initial BER (mg)] * 100 (**1**LC (%) = [Initial BER (mg) – Free BER in supernatant (mg) / NP-lyophilized form (mg)] * 100 (2 and SY (%) = [NPs lyophilized form of NPs (mg) / initial BER (mg) + initial BSA (mg)] * 100 (3, respectively:

1 $$\text{EE }\,(\mathrm{\%}) = [\text{Initial BER }(\mathrm{mg}) -\text{Free BER in supernatant }(\mathrm{mg})/\text{Initial BER }(\text{mg})] * 100$$

2 $$\mathrm{LC}\,(\text{\%}) = [\text{Initial BER }(\text{mg}) -\text{Free BER in supernatant }(\text{mg}) /\text{NP}-\text{lyophilized form }(\text{mg})] * 100$$

3 $$\text{SY}\,(\text{\%}) = [\text{NPs lyophilized form of NPs }(\text{mg}) /\text{initial BER }(\text{mg}) +\text{initial BSA}(\text{mg})] * 100$$

The BER release profiles of free BER and BER-BSA NP were studied for 48 h at two different pH values using 0.1 M phosphate buffered saline (PBS, pH 7.4) and 0.01 N HCl (pH 2.0), which is similar for simulated intestinal fluid (SIF) and simulated gastric fluid (SGF), respectively. The cumulative BER release rate (%) in the dialysis medium was calculated by using the following equation Cumulative BER release rate (%) = [Released BER (mg)/Initial BER (mg)] * 100 (4:

4 $$\text{Cumulative BER release rate}\,(\text{\%}) = [\text{Released BER }(\text{mg})/\text{Initial BER}(\text{mg})] * 100$$

Zetasizer Ver. 6.20 (Malvern Instruments Ltd., UK) was used to measure the hydrodynamic particle size diameter, polydispersity indices (PDI) and zeta potential of the BER-BSA Nps and BSA Nps where dynamic light scattering (DLS) / photon correlation spectroscopy was used with a particle seizer at a fixed angle of 90° at 25 °C. Furthermore, the morphological characterization and the size of BBR-BSA NPs and BSA NPs were estimated by field emission-transmission electron microscopy (FE-TEM) photomicrographs (JSM 1400 PLUS-JEOL). On the coated copper grid with the carbon file, drops of the suspended NPs were placed in deionized water and allowed to dry at room temperature before scanning at various magnifications.

### Animal design

Fifty male Wistar rats (*Rattus norvegicus*), weighing 125–135 g, were obtained from the Medical Research Institute—Alexandria University (Alexandria, Egypt). The animals were housed in polycarbonate cages and acclimatized to laboratory conditions for a period of one week prior to the start of the experiments. The rats were fed standard rodent food pellets (Agricultural‐Industrial Integration Company, Giza, Egypt) and double‐distilled water. The number of crude proteins, fats, and fibers in the food pellets was 12, 2.4, and 14%, respectively. The energy content of the standard diet was 920.48 kJ/100 g. All experimental procedures were performed according to the criteria outlined in the Guide for the Care and Use of Laboratory Animals approved by the Institutional Animal Care and Use Committees (IACUCs) of Pharmaceutical and Fermentation Industries Development Center, Scientific Research, and Technological Application City with approval number 21-1S-2220.

Liver hyperplasia was induced in rats by 5 consecutives intraperitoneal (IP) injection of 200 µg/kg/day AFB dissolved in dimethyl sulfoxide (DMSO) [12]. For treated groups, Both BER-BSA NPs and BER were dissolved in 5% Tween-80.

The animals were divided into five groups, each containing ten rats as follows:

- The sham control group that had no treatment.
- The Tween-80 control group which IP injected with 1 mL/kg DMSO for 5 days followed by orally administration of 1 mL/kg of 5% Tween-80 for one month [12].
- The induction group was IP injected with AFB1 for 5 days followed by oral administration of tween-80 for one month.
- BER-BSA NP-treated group: after the 5 consecutive IP injection with AFB1, this group was treated orally with BER-BSA NPs (10 mg/kg/day) for 30 days.
- BER-treated group, rats were treated with BER (100 mg/kg/day) for 30 days after the 5-day induction period [13].

After the treatment period (35 days), all rats fasted for 6 h. Then blood was drawn from the eye to measure the blood glucose level (BGL) [14] because it is well known that BGL must be measured during a fasting period of 6–8 h before the gluconeogenic process took place; then the rats completed the fasting period for 14 h. In an induction room, rats were subjected to 5% isoflurane (inhale); that because Isoflurane is a significant volatile substance that has been employed as rat inhalant anesthetic and has no effect on hepatic metabolism, making it appropriate for pharmacological and metabolic investigations; supplied with 95% oxygen at flow rate of 600 ml/min for 10 min. After completing rats’ anesthesia, the rats’ scarification process by decapitation was carried out [14] and this euthanasia process is run according to National Institute of Health guidelines. Blood and liver tissues were immediately collected. Where the blood was collected by heart puncture in a heparinized tube let to stand for 15 min at room temperature then centrifuged at 3000 rpm for 10 min to separate plasma. While, the liver was quickly isolated, washed in cold saline, and divided into three sections; the first section was used to prepare liver homogenate which was used to measure oxidative stress and inflammatory markers. This section was homogenized in a nine volume of 0.1 M sodium phosphate buffer saline, pH 7.4, then centrifuged at 3,000 rpm for 15 min at 4 °C, and finally the supernatant was collected.

The second part of the liver was submerged in an RNA-later stabilizing solution (Thermo Fisher Scientific, USA) then subjected to liquid nitrogen and stored at -80°C for one month after that liver specimen was collected and homogenized a total RNA extraction solution to isolate the total RNA to study gene expression.

The third part of the liver was fixed in a 10% neutral buffered formalin solution for histopathological examination.

### Routine blood parameter measurements

Blood glucose level, TG, cholesterol, ALT, AST, LDH, GGT, TP, ALB, T. Bil, AFP and CRP [13, 15] were measured using commercial kits (Spectrum, Egypt) according to the manufacturer's instructions.

### Oxidative stress marker measurements

Hepatic prooxidant parameters, including thiobarbituric acid reactive species (TBARS, μmol/mg protein), nitric oxide (NO, μg/mg protein) and nonenzymatic; GSH (μg/mg protein); and enzymatic antioxidant systems; glutathione S transferase (GST, μmol/mg protein), superoxide dismutase (SOD, U/mg protein) and catalase (CAT, U/mg protein); were measured according to Tappel & Zalkin, 1959, Montgomery and Dymock, 1961, Jollow et al*.,* 1974, and Aebi, 1984, respectively [16–19].

### Hepatic inflammatory marker measurements.

The hepatic levels of iNOS, COX-2, and arginase were measured using commercial sandwich ELISA kits (INOVA, China) according to the manufacturer’s instructions. All protein levels are expressed as ng/mg protein content.

### Quantitative real-time reverse transcription PCR analysis (qPCR)

Hepatic RNA was extracted using an Assay RedTM Total RNA extraction kit according to the iNTRON protocol. After measuring the RNA concentration and purity, one microgram was reversibly transcribed into cDNA by using the Maxime RT PreMix kit according to the iNTRON protocol. Specific primers for P53, PTEN, H19, IGF-2, and β-actin, as well as PCR conditions, are listed in Table 1. After mixing the cDNA template, primers, *Taq* qPCR green master mix (Vivantis, Malaysia), and nuclease-free water, the following thermal cycle conditions were applied: initial denaturation for 2 min at 95 °C, step 2, which consisted of several cycles (Table 1) of denaturation for definite at 95 °C for 15 s, annealing step as mentioned in Table 1 and extension step 60 °C. Gene expression was calculated using the 2^−ΔΔCt^ method, in which normalization was carried out by using β-actin as a housekeeping gene.

**Table 1:** Primer sequences and qPCR conditions

Gene name	Primer Sequence	Annealing Temperature (°C)	Number of Cycles	Reference
β -actin	F-5′- CTGACCGAGCTGGCTAC-3′ R-5′-CCTGCTTGCTGATCCACA-3′	53.4	35	[20]
P53	F-5′- CCTATCCGGTCAGTTGTTGGA-3′ R-5′-TTGCAGAGTGGAGGAAATGG-3′	57.3	35	[21]
PTEN	F-5′- TGAGTTCCCTCAGCCATTGCCT-3′ R-5′-GAGGTTTCCTCTGGTCCTGGTA-3′	63.5	40	[22]
H19	F-5′- TCAGCTCTGGGATGATGTGGT-3′ R-5′-CTCAGGAATCGGCTCTGGAAG-3′	62.5	40	[23]
IGF-2	F-5′- ATGTCACCCATGTCACCAAG-3′ R-5′-GGCTTGTGCCAATTAGGTTCT-3′	57	35	[24]

### Histopathological examinations

Hepatic samples were fixed in 10% neutral buffer formalin for histopathological analysis. After fixation, these tissues were embedded in paraffin. Solid sections were cut into 5–6 µm thick sections, which were stained with hematoxylin and eosin (H&E) for histological evaluations. These pancreatic sections were examined under light microscopy (Olympus, Tokyo, Japan), and their photomicrographs were taken (X200 or X400 magnifications). The semiquantitative scoring of aberrations that took place were analyzed by ImageJ 1.48v.

### Statistical analysis

All data are statistically expressed as means ± standard deviations of the means (means ± SD). Statistical significance (*p* < 0.05) of differences was evaluated using a one-way ANOVA with a post hoc LSD test of SPSS Windows Version 19.0 (SPSS, Inc., Chicago, IL, USA) for multiple comparisons. Heat map analyses were obtained from the ClustVis web server (https://biit.cs.ut.ee/clustvis/ accessed on 6 February 2022 [25]. 

## Results

**Fig. 1:**
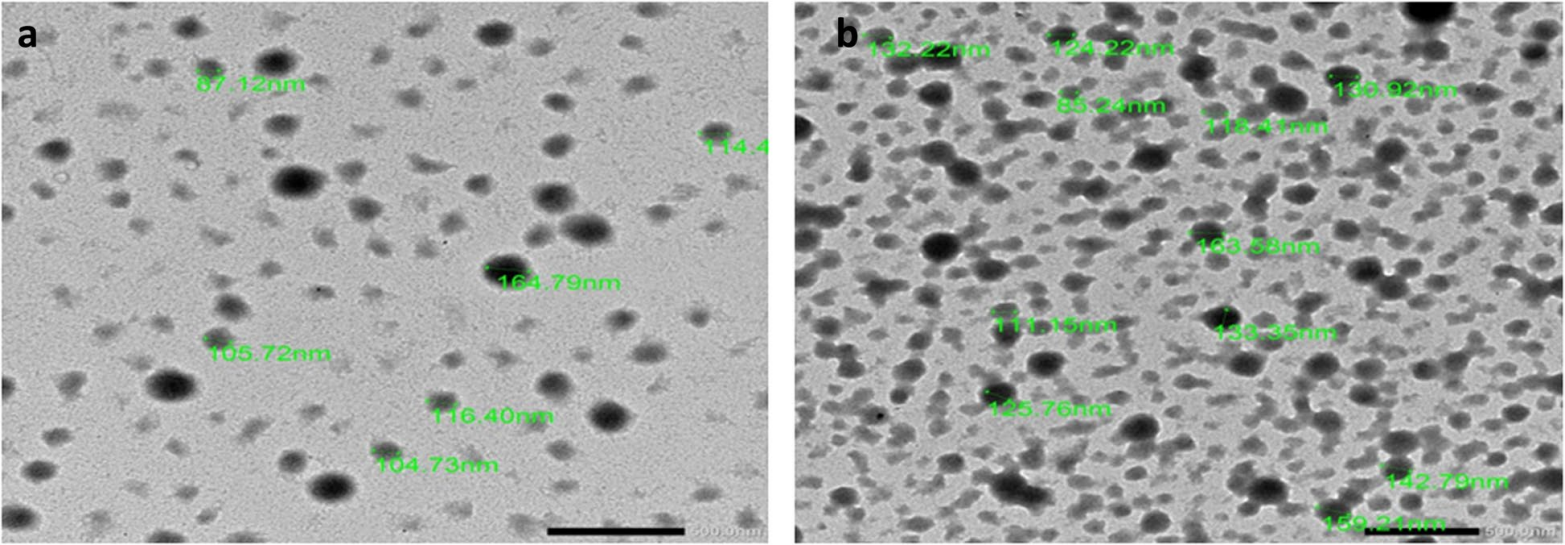
TEM photomicrographs of BSA NPs (**a**) at a magnification of 12,000 × and BER-BSA NPs (**b**) at a magnification of 10,000 × . The scale bar is 500 nm

Table 2 shows that the formed BSA NPs with 268 nm particle sizes and net positive charge (28.5 mV) were transformed into negatively charged NPs when BER was added, where the BER-BSA NPs with 320 nm particle sizes were homogeneously distributed (PDI was 0.413) with net negative charges, where they were 20.3 mV. The transmission electron microscope (TEM) image shows that both BSA and BER-BSA NPs had spherical, regular, and uniform shapes (Fig. 1).

**Table 2:** The particle size, polydispersity index, and zeta potential of berberine nanoparticles (BER-NPs)

Nanoparticles	Particle size (nm)	PDI	Zeta potential (mV)
BSA NPs	268 ± 2.5	0.321 ± 0.008	28.5 ± 0.99
BER-BSA NPs	320 ± 13.2	0.413 ± 0.003	-20.3 ± 1.12

**Fig. 2:**
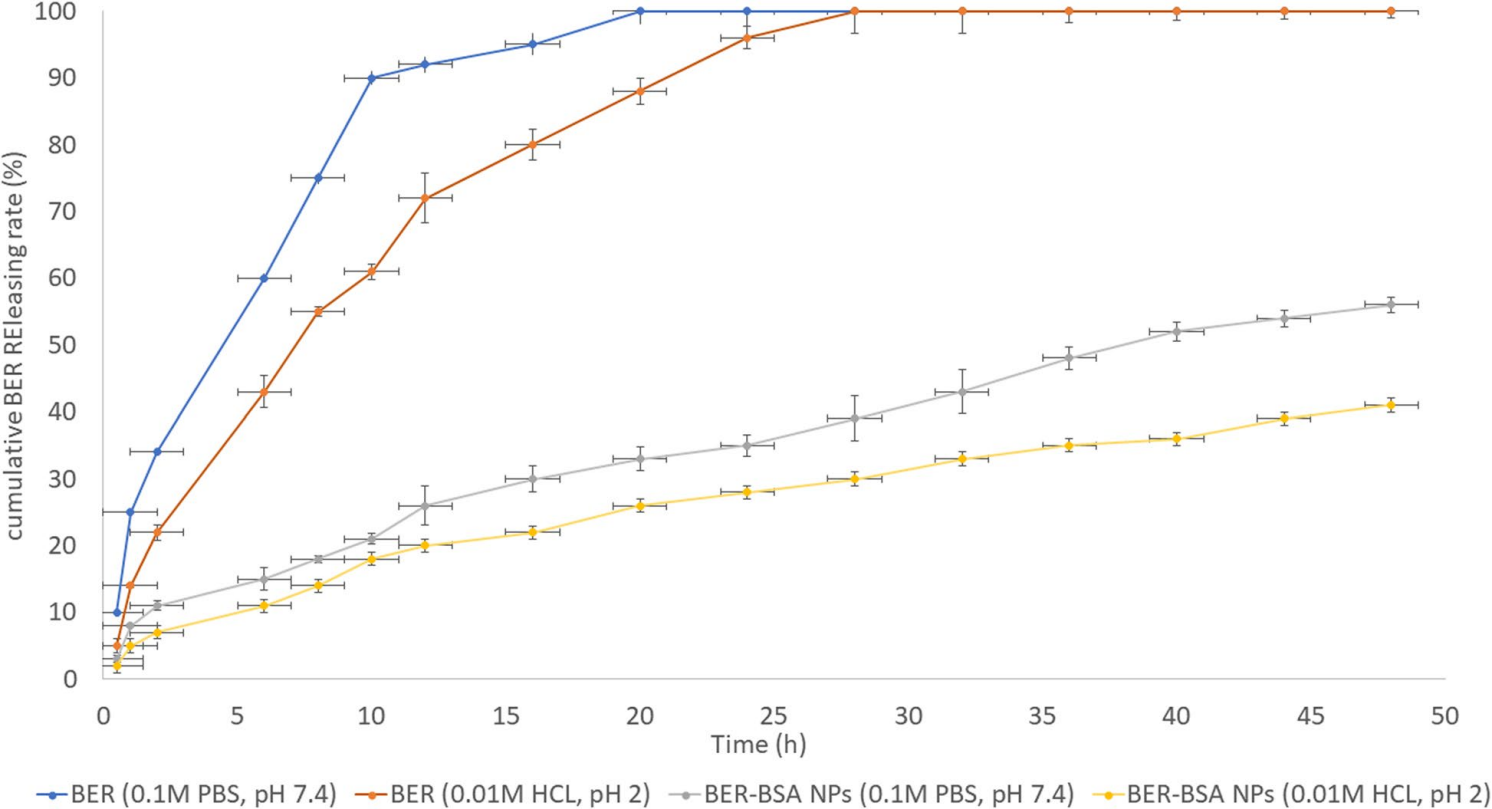
The BER-releasing models after 48 h. Data values are expressed as means ± SD (*n* = 5)

Table 3 shows that the% BER encapsulation efficiency was 78.5. The formed BER-BSA NPs showed a loading capacity % of 7.71 and a synthesis yield % of 92.6. Figure 2 shows that SIF stimulated BER resealing more than SGF medium. SIF enabled BER to 100 in 20 h, while SGF released 100% of BER after 28 h. In the case of BER-BSA NPs, the resealed BER % was 56% and 41% in the case of SIF and SGF, respectively, after 48 h.

**Table 3:** Ultraviolet–visible spectroscopic measurements of the synthesized BER-BSA and BSA NPs

Formulation	BER content (mg)	NPs content (mg)	EE (%)	LC (%)	SY (%)
BSA NPs	……	191.7 ± 2.7	……	….	95.8 ± 2.1
BER-BSA NPs	15.7 ± 0.1	203.7 ± 5.4	78.5 ± 1.7	7.71 ± 0.3	92.6 ± 1.8

Rats injected with Tween-80 showed the same control levels of plasma glucose, lipid profile, and liver enzyme activities, except that they showed higher LDH activity than the control, at *p* < 0.05. Rats that were administered aflatoxin for 28 days had hyperglycemia, hypercholesterolemia, hypertriglyceridemia, and hepatic injury where all liver enzyme levels (AST, ALT, ALP, LDH, and GGT) were markedly increased in plasma, at *p* < 0.05 when compared to control levels. Both treatments improved blood glucose levels, lipid profiles, and liver enzyme levels more than those of the induced group at *p* < 0.05. The therapy with BER-NPs was more efficient than the treatment with berberine, at *p* < 0.05, where it better improved the liver enzyme than that of berberine, and this treatment normalized AST and ALT levels, at *p* < 0.05, as shown in Table 4.

**Table 4:** Effect of different treatments on plasma levels of liver enzymes, glucose, cholesterol, and triglycerides

Groups/parameters	Glucose (mg/dL)	Triglycerides (mg/dL)	Cholesterol (mg/dL)	ALT (U/L)	AST (U/L)	ALP (U/L)	LDH (U/L)	GGT (U/L)
Sham control	144.01* ± 2.54	86.37^*^ ± 0.16	107.69^*^ ± 0.12	45.64* ± 0.35	49.14* ± 0.25	52.3* ± 3.25	62.9* ± 6.35	23.2* ± 2.35
Tween control	148.81* ± 0.26	81.97^*^ ± 0.12	109.73^*^ ± 0.14	43.24* ± 0.17	48.94* ± 0.11	58.2* ± 1.5	80.2#* ± 1.87	29.7* ± 0.95
Induction	278.24^#^ ± 0.23	450.73^#^ ± 1.51	192.86^#^ ± 0.25	96.45^#^ ± 0.07	120.55^#^ ± 0.35	275^#^ ± 8.5	600.5^#^ ± 12.4	80.1^#^ ± 8.7
BER treatment	180.8*^#^ ± 0.93	250*^#^ ± 3.8	168.64*^#^ ± 10.3	70.31*# ± 0.18	82.11#* ± 0.32	160.3#* ± 8.8	258.3#* ± 9.7	60.5#* ± 6.4
BER-BSA NPs treatment	160.9^*#&^ ± 1.93	200*^#&^ ± 6.9	150.97*^#&^ ± 3.6	44.18*& ± 0.12	48.27*& ± 0.26	100.3#*& ± 7.6	189.1#*& ± 6.7	43.2#*& ± 1.54

Data are represented as mean ± SD, and means with * are statistically significant at *p* < 0.05 when compared to the induction group, and means with # are statistically significant at *p* < 0.05 when compared to the control group. Mean with & is statistically significant at *p* < 0.05 when compared to berberine treatment

**Fig. 3:**
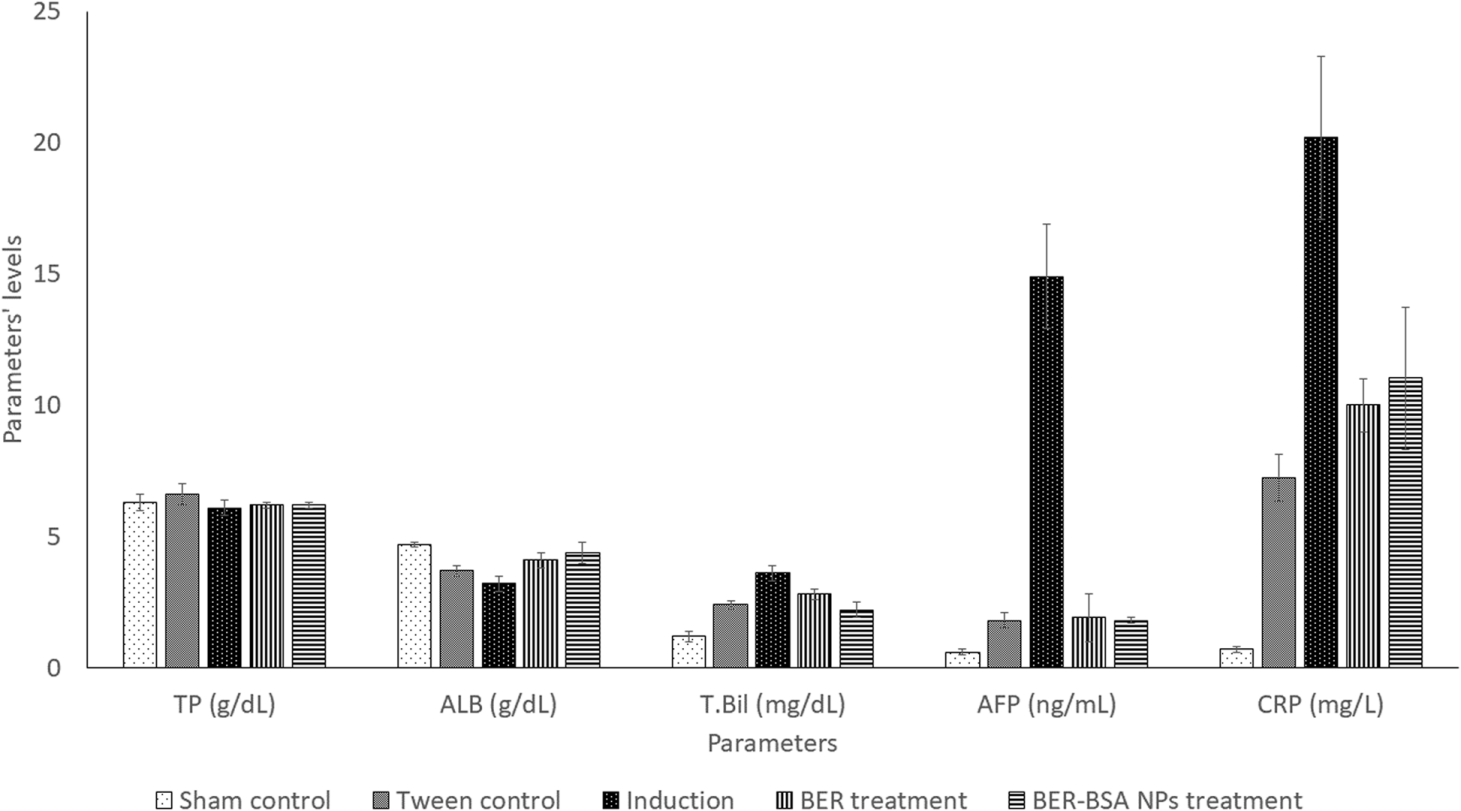
Effect of different treatments on plasma protein, albumin, bilirubin, alpha fetoprotein, and C-reactive protein

Figure 3 shows that none of the treatments altered the protein levels among all the groups, at *p* < 0.05. At the same time, the albumin level decreased after Tween treatment and aflatoxin administration, which showed the lowest albumin level, at *p* < 0.05. Both treatments increased the albumin level more than that of the induced group at *p* < 0.05, but treatment with BER-NPs normalized the albumin level. Tween-80 administration increased the total bilirubin level, AFP, and CRP compared with those of the control group at *p* < 0.05. Aflatoxin administration showed the highest values of T. Bil, AFP, and CRP, at *p* < 0.05. Both treatments improved the three parameters but failed to normalize them, at *p* < 0.05, but the treatment with BER-NP was more efficient than the treatment with berberine, at *p* < 0.05.

**Fig. 4:**
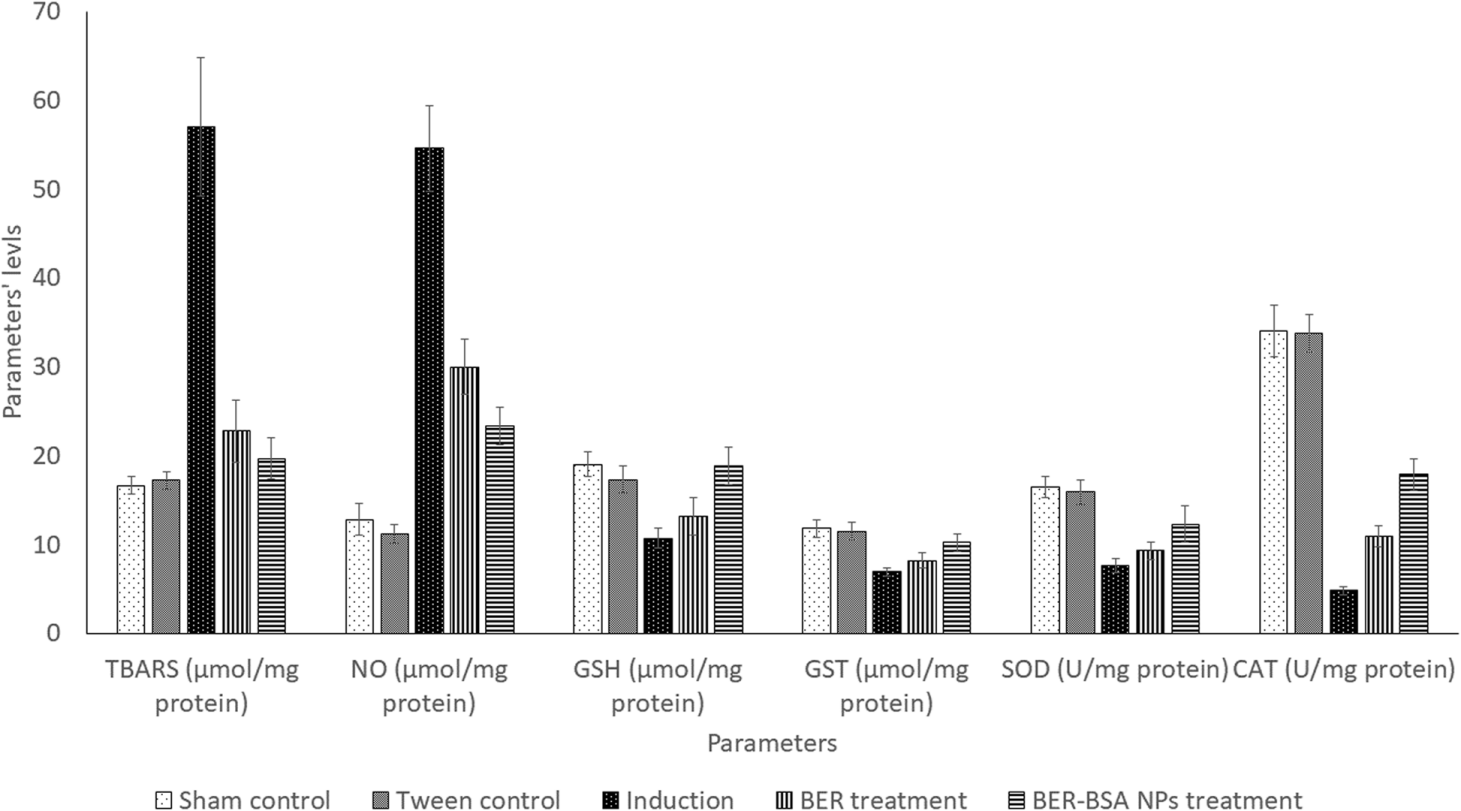
Effect of different treatments on hepatic prooxidants and antioxidant parameters

Aflatoxin administration led to hepatic oxidative stress, where it increased hepatic prooxidant levels (TBARS and NO) and decreased nonenzymatic antioxidant (GSH) and enzymatic antioxidant systems (GST, SOD, and CAT) when compared to sham control group levels at *p* < 0.05. On the one hand, berberine and BER-NP treatment improved the antioxidant system and decreased pro-oxidant parameters but did not normalize them, at *p* < 0.05. On the other hand, treatment with BER-NP normalized liver GSH and GST levels at *p* < 0.05; therefore, treatment with BER-NP was more efficient than treatment with berberine, as shown in Fig. 4. Moreover, aflatoxin administration increased hepatic iNOS, COX-2, and arginase levels, at *p* < 0.05. Both treatments improved the enzyme levels, but the treatment with BER-NPs was more efficient than the treatment with berberine, at *p* < 0.05 (Table 5).

**Table 5:** Effect of different treatments on liver inflammatory markers (iNOS and COX-2) and arginase levels

Groups/parameters	iNOS (ng/mg protein)	COX-2 (ng/mg protein)	Arginase (ng/mg protein
Sham control	2.05* ± 0.12	0.53* ± 0.05	3.48* ± 0.11
Tween control	2.12* ± 0.02	0.51* ± 0.01	4.45#* ± 0.35
Induction	4.34# ± 0.22	1.18# ± 0.09	0.47# ± 0.17
BER treatment	2.9#* ± 0.01	0.86#* ± 0.01	1.21#* ± 0.08
BER-BSA NPs treatment	2.34#*& ± 0.01	0.63#*& ± 0.03	2.32#*& ± 0.09

Data are represented as mean ± SD, and means with * are statistically significant at *p* < 0.05 when compared to the induction group, and means with # are statistically significant at *p* < 0.05 when compared to the control group. Mean with & is statistically significant at *p* < 0.05 when compared to berberine treatment

Tween 80 administration up-regulated P53, PTEN, and IGF-2 gene expression, which was associated with the downregulation of H19. Administration of aflatoxin down-regulated P53 and PTEN, which was associated with up-regulation of the expression of the H19 and IGF-2 gene. Compared to the induction group, both treatments up-regulated P53 and PTEN and down-regulated H19 and IGF-2 at *p* < 0.05. Treatment with BER-NPs was more efficient than treatment with berberine, at *p* < 0.05, as shown in Table 6.

**Table 6:** Effect of different treatments on the relative expression of p53 and BCL-2 in the liver

Groups/parameters	P53	PTEN	H19	IGF-2
Tween control	1.93* ± 0.013	5.20* ± 0.08	0.32* ± 0.06	1.13* ± 0.06
Induction	0.18* ± 0.03	0.11* ± 0.06	2.67* ± 0.17	3.54* ± 0.058
BER treatment	0.93* ± 0.011	2.62* ± 0.028	1.64* ± 0.05	2.11* ± 0.058
BER-BSA NPs treatment	1.05*& ± 0.03	3.47*& ± 0.11	0.09*& ± 0.01	1.27*& ± 0.07

Data are represented as mean ± SD, and means with * are statistically significant at *p* < 0.05 compared to the induction group. Mean with & is statistically significant at (*p* < 0.05) compared to berberine treatment

**Fig. 5:**
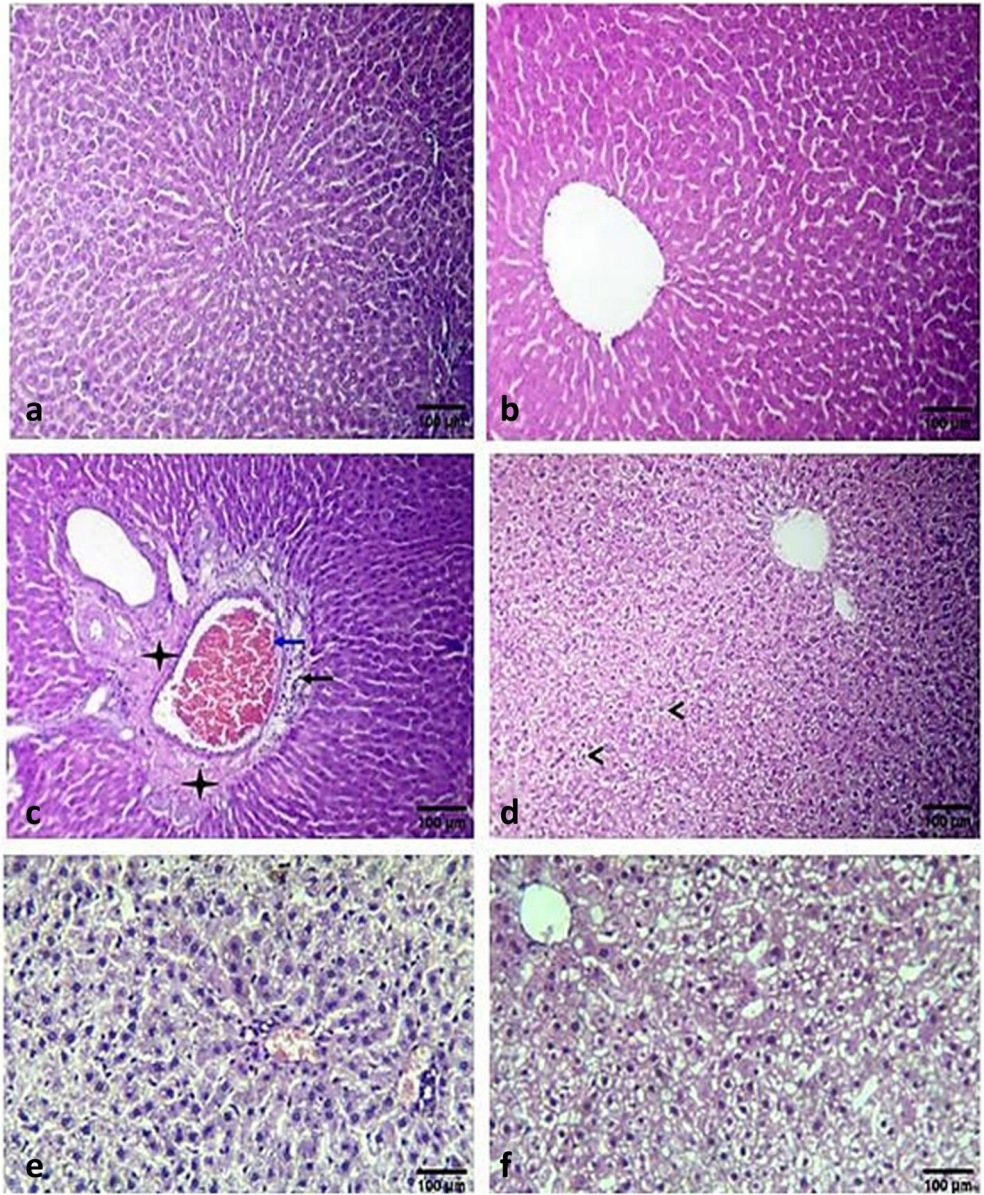
A photomicrograph of an H&E-stained T.S. in rat liver of the sham control (**a**), Tween-80 control (**b**), induction group (**c** and **d**), treated group with BER (**e**) and treated group with BER-BSA NPs (**f**). The images (**a**-**d**) were magnified at X200, while the images e and d were magnified at X400

Figure 5 and Table 7 show that both the control and the Tween control groups (Fig. 5a and b) had normal hepatic architecture, where images a and b show that normal hepatocytes are arranged in cords around the central vein. The induced group had congestion of the portal vein (blue arrow), mild inflammatory cell infiltration (black arrow), faint eosinophilic albuminous edema in the portal area (stars) (Fig. 5c), and vacuolar and hydropic degeneration (arrowheads) (Fig. 5d). The berberine-treated group showed mononuclear cell infiltration (Fig. 5e), while the BER-BSA NP-treated group showed a normal liver architecture like that of the sham control (Fig. 5f).

**Table 7:** Histological scores in liver

Groups	Sinusoidal dilatation	Vacuolar and hydropic degeneration	Hemorrhage	Mononuclear cells infiltration (Inflammation)	Necrotic hepatocytes
Control	+ +	-	-	-	-
Tween control	+ +	-	-	-	-
Induction (c-d)	-	+ + +	+ + +	+	+ + +
BER treatment	-	-	+ +	+ +	+
BER-BSA NPs treatment	-	+	-	-	-

− No histopathologic change

+ Histopathology in < 20% of fields

+  + Histopathology in 20 to 60% of fields

+  +  + Histopathology in > 60% of fields

## Discussion

Several studies have used BSA as a nanocarrier for bioactive compounds by the desolvation method because it has high selectivity, great bioavailability, and potent therapeutic properties [20, 26]. In this work, ethanol and glutaraldehyde were used as desolvation and crosslinking agents, respectively, in the desolvation method [21]. As PDI is a good indicator of NP homogeneity, a lower PDI value indicates a uniform distribution of NP in the medium [22]. The data presented in Fig. 1 and Table 2 indicate the incorporation of berberine on the surface of BSA-NPs, where the particle size increased and the zeta potential was converted from the positive charge in the case of BSA-NPs to the negative charge in the case of BER-BSA-NPs. The surface charge during formulation is also considered an indicator of the stability of the NP distribution, which indicates the degree of electrostatic repulsion between the particles. The highly charged particles have a high zeta potential value, which is stable due to the great electric repulsion forces between the molecules [22].

BSA is used in NP technology to increase the LC% and the biological properties of its entrapped bioactive ingredients [23]. Our study targeted encapsulating BER in the core of BSA NPs that delayed and minimized the BER release rates (%) in different dialysis media (SIF or SGF), which agrees with the report by Patra et al*.* in 2018 [24].

Therefore, BSA-NPs were used to reduce the therapeutic dose of BER, eliminate its local dissolution rate, induce its solubility, and promote its absorption activity. To examine this hypothesis, the therapeutic effect of BER-BSA-NP at a dose of 10 mg/kg compared to the recommended dose of BER (100 mg/kg) against AFB1-induced liver hyperplasia was measured.

AFB1 administration induced hepatotoxicity, which ranged from liver injury until HCC development [27]. The exact mechanism by which AFB1 induces liver lesions is still unclear, which limits the identification of specific therapeutic compounds for AFB1-related HCC. In this study, AFB1 administration induced oxidative stress, liver inflammation, proliferation, and invasion of cancer cells. Where AFB1 increases prooxidants markers (TBARS and NO), decreases antioxidants systems (GSH, GST, SOD and CAT), stimulates inflammatory enzymes (CRP, iNOS and COX-2), inhibits anti-inflammatory markers (ARG1), decreases the tumor suppressor enzyme (PTEN), increases oncogene (AFB), increases glycolytic activity (IGF2), prevents cell death (P53) and promotes cell growth (H19).

The liver transformation of AFB1 leads to the formation of free radical metabolites that interact with cellular biomolecules, leading to the formation of pro-oxidant molecules and suppression of non-enzymatic antioxidants (GSH), which consequently leads to the depletion of the enzymatic antioxidant system. This phenomenon indicates the existence of oxidative stress [27]. It is well known that oxidative stress activates inflammation. AFB1 promotes the expression of the inflammatory mediators COX-2 and iNOS [28, 29]. Both mediators stimulate cytokines, such as IL6, production from macrophages and adipocytes that trigger the synthesis of CRP and fibrinogen by the liver [30]. Arginase I, which is a liver enzyme responsible for the urea cycle that is secreted from activated macrophages and plays a central role in anti-inflammation and tumor immunity processes, is downregulated in HCC [31].

AFB1-induced oxidative stress suppresses the PI3K/AKT/mTOR system to stimulate autophagy [4]. Autophagy plays a key role in cancer cell survival during starvation (low ARG1) and oxidative stress conditions [32] to help the cell find alternative sources of ATP. Although PTEN is a negative regulator of the PI3K/AKT/mTOR pathway and must be increased during inhibition of this system to induce autophagy, PTEN plays an important nuclear tumor suppressor factor, as it maintains genome integration and repairs DNA double-strand breaks [33]. Therefore, low PTEN is associated with cancer progression. Mouse livers with HCC have been reported to have 20 times higher levels of IGF2, which are expressed in the cytoplasm, rough endoplasmic reticulum, and mitochondria of malignant hepatocytes [34]. Furthermore, it has been reported that IGF2 overexpression is associated with down-regulation of PTEN and H19 up-regulation [35]. IGF2 overexpression increased glucose consumption, increased lactate production, and increased the mRNA expression of glucose and lactate transporters and glycolytic enzymes involved in cancer development and progression [36].

Long noncoding RNAs (lncRNAs) have a wide range of biological functions, including cell proliferation, cycling, apoptosis, differentiation, and invasion. One of the most important lncRNAs expressed in AFB1-induced HCC is H19, which is found on chromosome 11p15.5 and also contains the IGF-2 gene in an upstream location; therefore, both genes are overexpressed. H19 expression is suppressed by P53 and vice versa [37]. As mentioned before, AFB1 induced autophagy, which is used as a survival mechanism. Autophagy and apoptosis can cross-inhibit each other. The inhibition of autophagy-induced apoptosis is mediated by the degradation of pro-apoptotic factors, for example damaged mitochondria and caspases, which play a pivotal role in intrinsic apoptosis and finally prevent p53 production [15]. Routine blood parameters and histological studies confirmed AFB1 toxicity, where hyperlipidemia, hyperglycemia, hepatic and nephrotoxicity occurred, which is in agreement with the report by Geo et al. [28].

Berberine and the berberine rich extract have been reported as hepatoprotective against AFB1 toxicity, where Berberis vulgaris ethanolic extract (200 mg/kg) that contained berberine as 60% of its contents protects the liver, as indicated by normal liver architecture and normal liver enzymes that occurred mainly due to the antioxidant properties of berberine that are reflected by increasing hepatic antioxidant and apoptotic parameters [38]. Furthermore, a diet supplemented with BER (600 mg/kg) increased meat quality and reduced oxidative stress in broilers fed an AFB1-contaminated diet [39]. Although BER acts as an inhibitor of the PI3K/AKT pathway and can induce autophagy, indicating that it is not a good therapeutic candidate for AFB1 toxicity, it has been reported that BER induces apoptosis through the activation of the mitochondrial pathway and inhibits autophagy through accumulation of LC-II, which inhibits the formation of autophagosomes [40]. Moreover, BER has been reported to dose-dependently upregulate PTEN [41]. Mice with nonalcoholic steatohepatitis that were treated with 50 mg/kg BER for 9 weeks showed down-regulated H19, high antioxidant levels, low lipid and glucose profiles and low inflammatory markers, indicating that BER decreased inflammation by preventing activation of Kupffer cells characterized by high ARG1, inhibiting hepatic stellate cell activation by inhibiting H19 and increasing insulin sensitivity by reducing IGF2 [42].

It is well known that this high dose is used because BER has a low absorption dose, as only 0.5% of ingested BER is absorbed in the small intestine, while this percentage is further decreased when it enters the systemic circulation. Additionally, the use of these high doses for treatment leads to several side effects, such as diarrhea, constipation, gas, and an upset stomach [9]. In agreement with our results, the results of Lam et al. [43] proved that the therapeutic activity of BER/albumin nanoparticles were achieved at doses of BER (1 mg/kg), while the same effect was observed at a BER dose of 10 mg/kg, which indicated that the nanoparticles exert their action at 1/10 of the active compound dose. Moreover, it has been reported that the in vitro cytotoxic effect of BER-BSA NPs against breast cancer is twice as high as that of pure BER [21].

## Conclusions

In the present study, we successfully developed/formulated a biocompatible, nontoxic, novel antiAFB1 toxicity berberine delivery system. BER-BSA NPs. The nanoparticles were synthesized by the desolvation method and characterized successfully. Particle Size and Morphology.

**Fig. 6:**
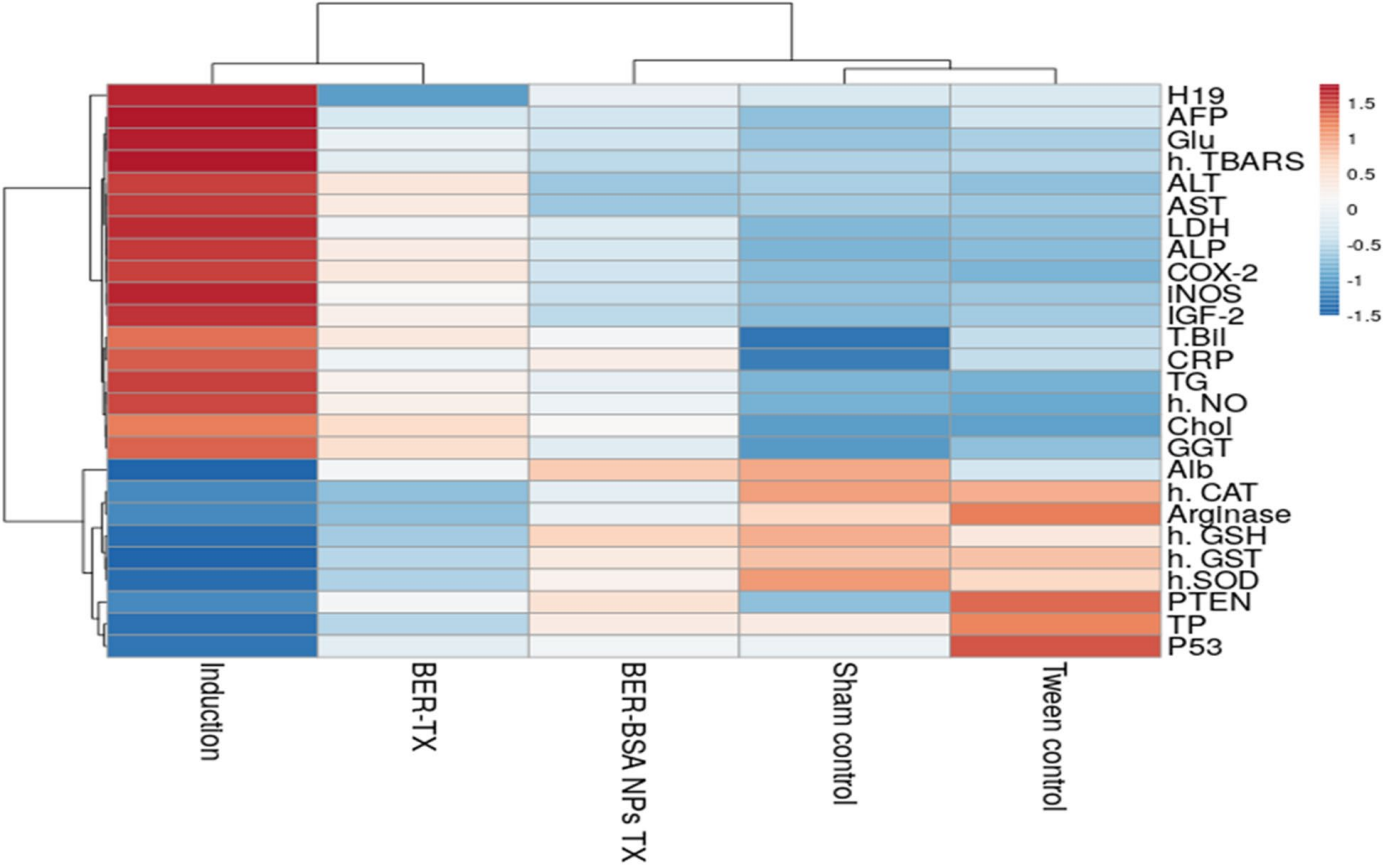
Heat map of the parameters measured in all tested groups (http://biit.cs.ut.ee/clustvis/). The red color indicates an increase in the values of the target parameters. The blue color demonstrates a decrease in the values of these parameters

The stability analysis indicated that the BER-BSA NPs were quite stable, spherical, and nearly monodispersed in nature. AFB1 induced liver toxicity by stimulating oxidative stress/autophagy/inflammation, glycolytic activity and cell growth associated with apoptosis prevention. BER-BSA NPs prevented all these events by inducing apoptosis and preventing autophagy at a dose of 1/10 of the pure BER dose, as shown in the heat map Fig. 6, which proved that BER-BSA-NPs were closer to normal control values.

## Data Availability

The datasets used can be accessed directly through the corresponding author (d.ghareeb@alexu.edu.eg) upon request.
